# The effect of forced even pacing and an opponent on end‐spurt behaviour in freestyle pool swimming

**DOI:** 10.1002/ejsc.12102

**Published:** 2024-04-08

**Authors:** Joshua E. Neuloh, Andreas Venhorst, Sabrina Skorski, Tim Meyer

**Affiliations:** ^1^ Institute of Sports and Preventive Medicine Saarland University Saarbrücken Germany

**Keywords:** competition, opponent, pacing strategy, sports performance, water

## Abstract

To investigate the effect of forced even pacing through virtual pacing assistance and an opponent in a competitive setting on end‐spurt behaviour in freestyle swimmers, including related physiological underpinnings. Twenty‐seven competitive swimmers and triathletes were recruited. There were four 1500 m freestyle trials: (i) familiarisation time trial, (ii) self‐paced time trial (STT), (iii) head‐to‐head competition time trial (CTT) and (iv) forced even pacing through virtual pacing assistance time trial (FET). Eventually, 12 swimmers met the criteria for the CTT and FET to be included in the analysis. Changes in end‐spurt behaviour, finishing time and physiological parameters (lactate, cortisol, noradrenaline and heart rate) were analysed using a linear mixed model with fixed effects for trials and a random effect for swimmer identity. A separate linear model was computed for competition outcome. The end‐spurt for each race was determined by means of an end‐spurt indicator (ESI; ESI > 0 greater end‐spurt). Swimmers demonstrated a significantly greater ESI in FET (+2.6; *p* < 0.001) and CTT (+1.4; *p* = 0.022) compared to STT. Blood lactate concentration in FET (+1.0 mmol L^−1^; *p* < 0.001) and CTT (+1.6 mmol L^−1^; *p* < 0.001) was significantly higher than in STT. Winners had a significantly greater ESI than losers in CTT (+1.6 and *p* = 0.005). Swimmers utilised a greater end‐spurt through metabolically optimal forced even pacing by virtual pacing assistance and in a head‐to‐head competition due a larger mobilisation of anaerobic reserves as indicated by greater blood lactate concentrations. Winners had a significantly greater end‐spurt than losers despite similar metabolic disturbances.

## Highlights

- Compared to a self‐paced time trial, swimmers in a forced even pacing trial by virtual pacing assistance and in a head‐to‐head competition execute a greater end‐spurt.
- The larger end‐spurt is associated with larger mobilisation of anaerobic reserves as indicated by greater blood lactate concentrations.
- Winners had a significantly greater end‐spurt than losers despite similar metabolic disturbances.

## 1 INTRODUCTION

The presence of an end‐spurt in 1500 m freestyle pool races has been well described previously (Lipinska et al., 2016; Mytton et al., 2015; Neuloh et al., 2020). Figueiredo et al. (2011) analysed the energy contribution of each 50 m lap during simulated competitions finding that the energy costs in the last lap was second highest after the start lap in 200 m freestyle. Still, a more even pacing strategy is considered energetically optimal in swimming (Thompson et al., 2004) due to the high restive forces of water. Even small changes in the swim velocity are accepted to be very critical as any increase in velocity raises energy expenditure substantially (Lipinska et al., 2016). However, Skorski et al. (2014) found that moderate manipulation through an opponent in 400 m freestyle swimming races had a positive impact in the final stages of the race (Skorski et al., 2014). Particularly, the winners and medallists in elite competitive events seem to be able to mobilise greater fractions of anaerobic reserves to outsprint their competitors during the final metres (Neuloh et al., 2020). The presence of a competitor shifts the focus towards beating the opponent rather than utilising the energetically optimal even pacing strategy to achieve the fastest possible finishing time (Abbiss et al., 2008; Foster et al., 1994; Mauger et al., 2012). As such, the primary goal of a swimmer during competitions is to achieve the best possible position, whilst achieving a personal best time may become a secondary objective (Menting et al., 2019). This might be especially true in important races, such as World championships or Olympic Games, where success is rated by the colour of the medal. To evaluate the end‐spurt scientifically, an ‘End‐spurt indicator’ (ESI; arbitrary units) was designed by Neuloh et al. (2020). To define an individual ESI per race and subject, the difference between the swim velocity in the last lap and the corresponding velocity of the middle part of the race is divided by the respective individual standard deviation of the swim velocity in the middle part of the race (For further details, see Neuloh et al., 2020). Swimmers typically perform an end‐spurt in 1500 m freestyle with the magnitude being affected by the anticipated finishing position and the importance of the race (Neuloh et al., 2020). Consequently, greater end‐spurts have been reported in head‐to‐head races in which success is determined by marginally performing better than the opponents (Neuloh et al., 2022) but only when the athletes believe they can beat the opponent (Crivoi do Carmo et al., 2022).

Multiple studies in cycling (Crivoi do Carmo et al., 2022; Konings et al., 2016; Konings et al., 2017; Venhorst et al., 2018b; Williams et al., 2015; Wilmore, 1968) and running (Tomazini et al., 2015) showed that an opponent is an essential determinant of pacing regulation. It was found that the presence of an opponent in cycling influenced end‐spurt behaviour with the magnitude of the end‐spurt depending on the performance goal (Crivoi do Carmo et al., 2022). For example, Corbett et al. (2012) observed an increased anaerobic energy supply (indicated by higher maximal blood lactate concentrations) when cyclists competed in virtual competitions against an opponent, which could not be mobilised in individual time trials. However, racing and losing against a faster opponent can negatively impact end‐spurt behaviour through decreased self‐efficacy and motivation in the final stages of the race (Crivoi do Carmo et al., 2022). Falling behind an opponent clearly is a negative affective event in goal‐striving associated with a greater endocrinological distress response as indicated using higher levels of cortisol and noradrenaline concentrations in losers compared to winners during head‐to‐head‐competitions (Venhorst et al., 2018b). Thus, the presence of opponents per se and the competition outcome are important factors in the decision‐making process to execute an end‐spurt (Hettinga et al., 2017; Konings et al., 2018; Renfree et al., 2014).

So far, the pacing literature in swimming has retrospectively analysed end‐spurt behaviour in 800 and 1500 m in competitions (Neuloh et al., 2022). The direct influence of forced even pacing through virtual pacing assistance (assumingly energetically optimal) and an opponent on end spurt behaviour in swimming is still unknown. Moreover, the physiological underpinnings of an end‐spurt considering differential responses in winners have not yet been investigated.

Therefore, the aim of the current study was to investigate the effect of forced even pacing through virtual pacing assistance and an opponent on swimmers' end‐spurt behaviour, including related physiological underpinnings. It was hypothesised that forced even pacing through virtual pacing assistance and an opponent leads to a greater end‐spurt compared to an individual time trial due to greater mobilisation of anaerobic energy reserves. Furthermore, it was hypothesised that the head‐to‐head competition would elicit a greater stress response and that winners would demonstrate an absolute (and relative to a self‐paced time trial [STT]) greater end‐spurt than losers.

## 2 METHODS

### 2.1 Participants

Twenty‐seven competitive swimmers and triathletes (11 female; age: 18 ± 2.54 years, height: 181.6 ± 7.8 cm and weight: 70.2 ± 8.20 kg) were recruited. All participants were highly trained and competed at the national level in their respective sport (mean: 549.90 ± 81.25, min: 476.37 and max: 650.99 Swimming Points by World Aquatics for their 1500 m freestyle personal best time) corresponding to Tier 3 in a novel participation classification framework of McKay et al. (2022).

Out of 27 swimmers, six participants dropped out at various points during the study due to injury or illness. Therefore, only 21 swimmers completed all trials. Due to strict matching and performance criteria, 9 swimmers were excluded and eventually 12 swimmers (11 pool swimmers and one triathlete) met the criteria for the head‐to‐head competition and the forced even pacing time trial (FET) and were subsequently included in the analysis. To ensure a competitive environment during the end‐spurt, pairs with a gap of more than one body length (i.e. ∼1.7 s) apart at the 1300 m turn were excluded. During the FET, athletes who were more than 10.5 s behind the set pace at the 1450 m turn have also been excluded to ensure that the swimmer followed the pre‐set even pacing pattern. This benchmark is based on a mean difference of 10 s between first and third finishing place at the World Championships 2003–2019 in 1500 m freestyle (Lara et al., 2021). Anthropometric and performance data of swimmers included in the study can be found in Table 1. All participants provided prior written informed consent to the procedures used in this study, which were approved by the local ethics committee (Kenn‐Nr. 34/21) and carried out in accordance with the Declaration of Helsinki.

**TABLE 1:** Anthropometric and performance level data.

	Male (*n* = 8)	Female (*n* = 4)
Anthropometric data		
Age (years)	17 ± 2	17 ± 3
Stature (cm)	184 ± 9	171 ± 5
Body mass (kg)	74.8 ± 7.8	60.6 ± 4.5
Performance level data		
Personal best time (min:ss.hh)	17:39.41 ± 48.86	20:44.17 ± 109.79
Swimming Points	561.91 ± 70.00	420.90 ± 109.66

### 2.2 Study design

All participants completed four trials with 1 week between sessions. The first trial was a baseline STT for familiarisation (FAM). Thereafter, swimmers completed three 1500 m trials in set order: a STT, a head‐to‐head competition time trial (CTT) and a FET using the Virtual Swim Trainer System (VST). The FAM test acted as a familiarisation time trial (FAM), and the STT was a STT without an opponent. The fastest of both finishing times (FAM and STT) was used to match swimmers for the CTT to ensure that swimmers were matched on their best performance capabilities. The finishing times and matches were not shared with the participants until the start of the CTT. The finishing time from CTT was the evenly distributed set pace for FET. Accordingly, no randomisation of the trial sequence was possible. Swimmers were asked to log their training, recovery training and diet before the first trial and replicate it accordingly for subsequent trials. All trials were completed at the same time of day to minimise diurnal biological variation (in assessed physiological parameters). The participants have been advised to reduce the training load 48 h prior to each session and prepare in the same manner as they would for a competition. The warm‐up before the trials consisted of 800 m self‐selected training followed by an ∼20 min delayed start. The participants completed all trials in freestyle, as this is the only stroke offered in swimming competitions for 1500 m.

### 2.3 Session 1—FAM

In session 1, swimmers completed a 1500‐m freestyle solo swim from a push start. The push start has been used to ease following the pacing lights from the start, and therefore, all time trials have been started with the same technique.

### 2.4 Session 2—STT

In session 2, swimmers completed a 1500‐m freestyle solo swim from a push start equal to session one.

### 2.5 Session 3—Head‐to‐head CTT

In session 3, swimmers completed a 1500 m head‐to‐head competition. To ensure a head‐to‐head‐competition with uncertain outcomes, swimmers were matched as closely as possible in pairs based on the fastest finishing time of the slower swimmer from FAM and STT. The mean difference of all pairs was 5.92 ± 6.30 s (maximum 19.56 s, minimum: 0 s). This mean difference is considerably lower than the mean difference of 10 s between first and third finishing place at the World Championships 2003–2019 in 1500 m freestyle (Lara et al., 2021). The time was programmed into the VST (Indico Technologies; see below) evenly and both swimmers followed the lights for the first 1300 m. For the last 200 m, the VST was stopped and both swimmers were encouraged to race each other and beat the opponent without any pacing feedback. The separation of 200 m was based on previous findings revealing the lowest mean swim velocity at around 1300 m and leaving enough room for tactical considerations by the swimmers before the end‐spurt (Neuloh et al., 2022). As a secondary goal, swimmers were encouraged to finish the trial in the shortest possible time independent of the head‐to‐head competition outcome.

### 2.6 Session 4—FET

In session four, swimmers had to complete a 1500 m trial with a forced even pacing. The pace was set based on their finishing time from CTT with the VST being programmed to distribute the CTT finishing time evenly among the 1500 m. This trial was conducted as a solo swim. Swimmers were encouraged to keep up with the target light until 1450 m without falling behind the LED lights by more than one body length and were then allowed to out sprint the pacing lights on the last 50 m if possible.

### 2.7 Virtual Swim Trainer system

The VST (Indico Technologies) was used in the CTT and FET trials. The light feedback system comprised of waterproof LED lights placed on the bottom of the pool throughout 50 m in the middle of the swim lane. The LED stripe was held down by small weights placed every ∼4–5 m. The lights have been pre‐programmed through the Swim Session Creator (Indico Technologies) and imported into the control panel (VST 17) of the Virtual Swim Trainer. For all testing sessions, including the lights condition, the LED stripes were programmed for even pacing on all 50 m split times according to the length of the pool (50 m). Every turn was indicated by a static white light 1.5 m before the wall and every push‐off the wall by a static white light by 7 m after the wall. The light feedback through the turn was set for all swimmers at 1.5 s for the spin and 3.6 s for the push‐off, which is the standard selection in the VST for competitive swimmers and in accordance with previous findings (Morais et al., 2019; Weimar et al., 2019).

### 2.8 Measurements

Before the warm‐up of the first trial, each participant had their age and body mass recorded. During each time trial, split times were taken every 50 m using handheld stopwatches (Interval 2000, Nielsen Kellermann) by skilled swim coaches to ensure the pacing was precise and to account for individual end‐spurt behaviour.

Heart rate was measured constantly through Polar OH1 optical heart rate sensor (Polar Electro Oy), which was placed under the swim cap at the temple. Heart rate data were collected from all sessions and stored in the internal memory of the devices. After completion of each session, data were uploaded to Polar Flow (Polar Electro Oy) and then exported to Microsoft Excel (Microsoft software, Microsoft Corporation).

Venous blood samples from a superficial antecubital vein were taken in a supine position for (a) cortisol 1 min before swim start at rest and 15 min after each trial, (b) noradrenaline 1 min before swim start at rest and 1 min after each trial and (c) haemoglobin 1 min before swim start at rest and 1 min after each trial. The blood samples were placed into pre‐chilled vacutainers containing K_2_‐ethylenediaminetetraacetic acid for the analysis of noradrenaline and red blood cell count and serum clot activator for the analysis of cortisol. Where appropriate, samples were inverted four times, immediately centrifuged at 3000 rpm for 10 min, plasma/serum pipetted off and kept cool until stored at −80°C for subsequent analysis. Noradrenaline was analysed by means of a chemical and enzymatic derivatisation linked to an immunosorbent assay (2‐Cat ELISA) (Beckmann Coulter). Cortisol was determined through chemiluminescence immunoassay using an Access 2 (Beckmann Coulter). The degree of haemoconcentration was calculated, and all blood samples were subsequently corrected for plasma volume changes (Dill et al., 1974). Capillary blood (5 μL) was collected from an earlobe 1 min before the start and 3 min after each trial to determine peak lactate concentration and placed into a glass capillary (20 μL), which was then stored in a container with haemolysing solution. Subsequently, blood lactate concentrations (mmol L^−1^) were analysed through an enzymatically amperometric procedure using Super GL (Greiner DiaSys).

### 2.9 Statistical analysis

Statistical analyses were conducted using SPSS 21 (IBM). Data were tested for normality, equality of variances, equality of covariance matrices and sphericity. When the assumption of sphericity was violated, Greenhouse–Geisser correction was performed. Results are reported as mean ± *SD* and α‐error of *p* < 0.05 was accepted as the level of significance. Changes in ESI, finishing time and physiological parameters (lactate, cortisol, noradrenaline and heart rate) were analysed using a linear mixed model with fixed effects for trials (three levels: STT, CTT and FET) and a random effect for swimmer identity. Due to the limited sample size, a separate linear model was computed with winner/loser (based on outcome of CTT) to further isolate the difference found and investigate interaction effects. A calculation of effect size was made using Cohen's *d* with thresholds of ≤0.50 for small, ≥0.50 for medium and ≥0.80 for large. The sample size estimation revealed that 41 participants will be necessary to observe a significant interaction effect (two‐tailed alpha of >0.05, *β* > 0.80 and *d* = 0.41, G*Power, Version 3.1.9.7). This calculation is based on the effect size found between medallists (finishing place 1–3) and non‐medallists (finishing place 4–8) in ESI at finals at World Championships and Olympic Games (Neuloh et al., 2020). The limited resources were the primary reason (limited access to national and international level swimmers) for the choice of the sample size collected (Lakens, 2022).

## 3 RESULTS

### 3.1 End‐spurt indicator

The main effect on ESI was significant (see Table 2 for all data; *F* = 11.0 and *p* < 0.001). The ESI in FET and CTT was significantly higher compared to STT with large effect sizes ([*p* < 0.001 and *d* = 1.90] and [*p* = 0.022 and *d* = 1.12], respectively). The ESI in the FET and CTT was on average 2.65 and 1.40 higher than in the STT, respectively. The ESI in FET was 1.25 higher compared to CTT. This difference was significant (*p* = 0.037 and *d* = 0.079). The interaction effect of trial and CTT outcome (winner/loser) was significant (*F* = 6.0, *p* < 0.001) with winners showing a significantly greater ESI (+1.68) than losers in CTT (*p* = 0.005 and *d* = 1.44). Losers had a significantly higher ESI (+1.70) in FET compared to CTT (*p* = 0.018 and *d* = 1.08), whereas winners showed no significant differences in ESI between the two trials (+0.36, *p* = 0.701 and *d* = 0.26).

**TABLE 2:** Summary of result in all sessions, main effects and haematological measurements.

(*n*)	STT	CTT	FET		Main effect	*p* value^a^	Cohen's *d*
	12	12	12				
ESI	0.93 ± 0.95	2.33 ± 1.48	3.58 ± 1.65	STT/CTT	<0.001	0.022	1.12
	102.15 (CV)	63.52 (CV)	46.08 (CV)	STT/FET		<0.001	1.90
				CTT/FET		0.037	0.79
Finishing time (mm:ss.hh)	18:29.71 ± 1:40.34	18:29.21 ± 1:33.66	18:26.06 ± 1:36.72	STT/CTT	>0.600	‐	‐
	9.04 (CV)	8.44 (CV)	8.74 (CV)	STT/FET			
				CTT/FET			
Heartrate Ø	168.60 ± 24.89	161.75 ± 29.44	162.26 ± 28.43	STT/CTT	>0.279	‐	‐
	14.76 (CV)	18.20 (CV)	17.52 (CV)	STT/FET			
				CTT/FET			
Lactate (mmol L^−1^)	4.24 ± 1.81	5.19 ± 1.41	5.26 ± 1.64	STT/CTT	<0.001	<0.001	0.58
	42.69 (CV)	27.17 (CV)	31.18 (CV)	STT/FET		0.008	0.04
				CTT/FET		0.076	0.59
Cortisol (µg/dL)	6.23 ± 3.00	8.70 ± 3.24	7.18 ± 2.69	STT/CTT	>0.07	‐	‐
	48.15 (CV)	37.24 (CV)	37.47 (CV)	STT/FET			
				CTT/FET			
Noradrenaline	3.43 ± 1.31	2.95 ± 1.03	2.62 ± 1.22	STT/CTT	>0.205	‐	‐
	38.19 (CV)	34.92 (CV)	46.56 (CV)	STT/FET			
				CTT/FET			

Abbreviations: CTT, competition time trial; CV, coefficient of variance; ESI, end‐spurt indicator; FET, forced even pacing time trial; STT, self‐paced time trial.

^a^ Post hoc test.

### 3.2 Finishing time

Total times from all sessions are shown in Table 2. There was no significant difference in finishing time between trials (*F* = 0.5, *p* = 0.600). The best mean finishing time was reported in FET with a difference of 3.66 s to STT and 3.15 s to CTT. The mean difference between STT and CTT was 0.51 s.

### 3.3 Haematological data

The main effect on blood lactate concentration was significant (*F* = 11.5 and *p* < 0.001). The blood lactate concentrations in FET and CTT were significantly higher than in STT. The difference between FET and STT was 1.01 mmol L^−1^ (*p* < 0.001; *d* = 0.04) and between CTT and STT 1.67 mmol L^−1^ (*p* < 0.001; *d* = 0.58). There was no significant difference between FET and CTT (0.65 mmol L^−1^ and *p* = 0.118). We found a significant interaction effect between trial and winner/loser (*F* = 4.6 and *p* < 0.001). On average, swimmers had a significantly higher blood lactate concentration in CTT than in STT (*p* = 0.003) and FET (*p* = 0.010), respectively. There was no significant difference between winner/loser in CTT (*p* = 0.216). The main effect on heart rate (*F* = 1.4 and *p* = 0.279), cortisol (*F* = 3.0 and *p* = 0.073) and noradrenaline (*F* = 1.7 and *p* = 0.205) was not significant. Although it did not reach the significance level (*p* = 0.07), blood cortisol concentrations were 2.47 µg/dL higher in CTT compared to STT (*d* = 2.74).

## 4 DISCUSSION

The main findings of the present study were (i) swimmers execute a greater end‐spurt through forced even pacing by virtual pacing assistance and in the presence of an opponent as compared to self‐pacing, (ii) this is paralleled with a larger mobilisation of anaerobic reserves as indicated by greater blood lactate concentrations and (iii) winners had a significantly greater end‐spurt than losers despite similar metabolic disturbances.

Swimmers showed a greater end‐spurt through forced even pacing by using virtual pacing assistance and predicted greater tolerance of metabolic disturbance, which is supported by higher blood lactate concentrations. Swimmers were guided to evenly pace the first 1450 m and then outsprint the pacing lights where possible. As there was no competitive opponent in FET opposed to CTT, we suggest that swimmers were able to perform an end‐spurt and access the anaerobic reserves to a greater extend by profiting from a more energetically optimal even pace in the first stages of the trial. It is generally accepted that mathematically and energetically an even pacing pattern in 1500 m freestyle swimming is optimal (McGibbon et al., 2018) and even small variations in the pacing strategy may have a substantial influence on the performance outcome (de Koning et al., 1999). Looking at the energy expenditure during virtual even pacing assistance in cycling, it was found that athletes had a higher power output assisted by greater anaerobic energy contribution in the end‐spurt, whereas the aerobic energy yield remained unchanged (Corbett et al., 2012). Foster et al. (2003) also showed that cyclists reserve some anaerobic energy during simulated competition, and it is understood that athletes monitor their energetic resources in a manner designed to optimise performance outcome. We propose that there is a positive effect through forced even pacing by virtual pacing assistance influencing the degree of metabolic stress that can be tolerated by swimmers.

Another argument could be the focus on achieving a better finishing time rather than beating an opponent. The external guidance (through the VST) can be assumed to represent a motivational factor for the swimmer, where the knowledge of being able to swim at the set pace and visual confirmation of it improves end‐spurt (Corbett et al., 2012). Although there are no supporting studies in swimming, it can be suggested from our findings and studies in other sports (Noakes et al., 2004; Williams et al., 2015) that forced even pacing by virtual pacing assistance through the VST also could have a motivational effect. In cycling, it was found that a simulated avatar serving as an opponent but actually representing the fastest previous performance of the athlete, improved time trial performance and end spurt (Williams et al., 2015). It was suggested that an increase in motivation positively influenced the willingness to exert the required effort, tolerate the associated physical discomfort of intensified performance and overcome negative factors, such as fatigue (Noakes et al., 2004; Williams et al., 2015).

It is generally accepted that athletes perform better in head‐to‐head competitions (Tomazini et al., 2015; Williams et al., 2015; Wilmore, 1968) than when exercising alone. The finding that swimmers showed a greater end‐spurt, while swimming in a head‐to‐head competition compared to a self‐paced trial is also in agreement with a recent study by Neuloh et al. (2020), who found that the end‐spurt is associated with the finishing position and that medallists performed a more pronounced end‐spurt than non‐medallists (Neuloh et al., 2020). As suggested by Corbett et al. (2012), the anaerobic energy reserve seems to be mobilised to a higher degree during competitions and needs to be accompanied by a greater tolerance of the metabolic stress (i.e., higher blood lactate concentrations) and associated physical discomfort. Konings et al. (2018) suggested that the behaviour of opponents is an essential determinant in the regulation of exercise intensity. It was shown that the competitive environment and the current internal state of the athlete influence the pacing behaviour related to an opponent. Thus, the decision‐making process of the athlete to elicit an end‐spurt or not is underpinned by biopsychosocial interactions.

In the current study, two swimmers were competing head‐to‐head against a closely matched competitor. Given an uncertain outcome as indicated by the (trend for a) higher blood cortisol concentrations in the CTT, both swimmers were motivated to change their pacing strategy from even to an end‐spurt and outsprint the opponent to win the race. The main aim in the CTT was to beat the opponent. It can reasonably be assumed that the winner in CTT felt motivated by being ahead and losers demotivated by the prospect of losing the race. Accordingly, winners were willing to access anaerobic reserves to a greater extent (as indicated by higher blood lactate concentrations) resulting in a greater end‐spurt compared losers and their own STT.

On the other hand, falling behind a performance matched competitor can clearly be conceived as a demotivational and negative affective event in goal‐striving in competitive athletes (Venhorst et al., 2018b). Accordingly, it has been shown that losers of head‐to‐head competition disengage from their initially set goal of winning and settle for a lesser goal, such as merely finishing the race (Crivoi do Carmo et al., 2022). This also explains the slightly better mean finishing time of all swimmers in FET compared to CTT as the motivational effect of winning is diminished by the demotivational effect of losing. Accordingly, Crivoi do Carmo et al. (2022) found that the presence of an opponent did not change overall performance, but differentially influenced pacing behaviour depending on the perceived outcome of winning or losing a race against an opponent and the respective maintenance or loss of self‐confidence.

## 5 LIMITATIONS AND SCOPE

The main limitation of the current study is the sample size as it is inherently difficult to recruit national level competitive swimmers. Due to dropouts and the application of strict matching criteria to ensure a competitive environment, all swimmers being more than 1.7 s behind the opponent in the CTT or 10.5 s behind the pacing light in FET were excluded from the analysis further reducing sample size. Moreover, the problem of imperfect matching of swimmers cannot be evaded. This is highlighted by the fact that winners in CTT also have been the swimmers with the faster time in FAM or STT, though there is always going to be one swimmer with a faster best time going into a matched head‐to‐head race. To prevent the confounder that swimmers may have changed their performance outcome in FAM or STT based on tactical reasons (to receive a slower opponent in CTT) was prevented by not sharing results and matching criteria. In fact, the trend for higher blood cortisol concentrations in CTT is indicative of a heightened stress response due to uncertain competition outcomes. The greater end‐spurt in FET compared to CTT is due to the fact of comparing group means, where losers had a lower or no end‐spurt in CTT, which affects the group mean of CTT when compared to FET. Losers were tapering off in CTT once they realised that they will lose.

It is generally accepted that a race against an opponent leads to greater motivation and that losing against a performance matched opponent is a demotivational and distressful event (Venhorst et al., 2018a). Given the objective difficulties in assessing motivational aspects through questionnaires or scales in swimming, there was no data collection on psychological aspects.

## 6 CONCLUSION

This study investigated the influence of forced even pacing through virtual pacing assistance and an opponent on end‐spurt behaviour and its physiological underpinnings. Swimmers performed a greater end‐spurt through metabolically optimal forced even pacing by virtual pacing assistance and in the presence of an opponent due a larger mobilisation of anaerobic reserves as indicated by greater blood lactate concentrations. Winners had a significantly greater end‐spurt than losers despite similar metabolic disturbances.

## CONFLICT OF INTEREST STATEMENT

No potential conflict of interest was reported by the authors(s).
